# Joint associations of cardiovascular polygenic risk and a health-related risk profile with incident cardiovascular disease among adults with depression: a UK Biobank study

**DOI:** 10.3389/fendo.2026.1934597

**Published:** 2026-08-20

**Authors:** Yohwan Lim, Ryuk Jun Kwon

**Affiliations:** 1 Gochang-gun Public Health Center, Ministry of Health and Welfare, Gochang, Republic of Korea; 2 Department of Biomedical Informatics, CHA University School of Medicine, Seongnam, Republic of Korea; 3 Family Medicine Clinic and Research Institute for Convergence of Biomedical Science and Technology, Pusan National University Yangsan Hospital, Yangsan, Republic of Korea; 4 Department of Family Medicine, Pusan National University School of Medicine, Yangsan, Republic of Korea

**Keywords:** cardiovascular disease, cohort study, depression, polygenic risk score, risk factor

## Abstract

**Background:**

Adults with depression have higher cardiovascular disease (CVD) risk, but the joint associations of inherited CVD susceptibility and health-related risk profiles remain uncertain. We examined incident CVD according to CVD polygenic risk score (PRS) and a health-related risk profile among adults with depression.

**Methods:**

We identified 3,446 participants with depression. For participants with ICD-10 depression recorded before recruitment, the index was the recruitment assessment date. For those identified by a Patient Health Questionnaire-9 (PHQ-9) score ≥ 10, the index was the questionnaire assessment date. CVD PRS was standardized and categorized into low, intermediate, and high tertiles. A health-related risk profile was constructed from current smoking, frequent alcohol consumption, obesity, low physical-activity frequency, and insomnia; 0–1 factors defined a favorable profile, and at least two factors defined an adverse profile. Cox proportional-hazards models assessed incident CVD during years 0–5. Years 5–10 landmark analysis included 3,062 participants who were alive, CVD-free, uncensored, and under observation at 5 years.

**Results:**

During years 0–5, 304 incident CVD events occurred. Compared with low PRS plus a favorable profile, high PRS plus a favorable profile was associated with higher CVD risk (adjusted hazard ratio [aHR], 2.00; 95% confidence interval [CI], 1.15–3.47). During years 5–10, 192 events occurred. Intermediate PRS plus an adverse profile (aHR, 2.02; 95% CI, 1.08–3.77) and high PRS plus an adverse profile (aHR, 1.89; 95% CI, 1.01–3.54) were associated with higher risk than low PRS plus a favorable profile.

**Conclusions:**

Inherited susceptibility was more evident in near-term CVD risk, whereas adverse health-related risk profiles were more evident in later risk among participants with intermediate or high PRS. These descriptive differences may provide complementary temporal risk information but do not establish that the relative importance of inherited and health-related factors changed over time.

## Introduction

Depression is increasingly recognized as an important risk factor for cardiovascular disease (CVD). Many studies have shown that depression is associated with a higher risk of CVD (1–3). In addition, polygenic risk scores (PRS) can stratify individuals according to genetic predisposition, but genetic risk is not proven to be deterministic (4–6). In previous studies, favorable lifestyle was associated with substantially lower coronary disease risk even among individuals with high genetic risk (5, 6). However, these findings do not necessarily imply that health-related factors neutralize genetic susceptibility, and evidence remains limited among adults with depression. This population frequently has smoking, physical inactivity, obesity, sleep disturbance, and cardiometabolic conditions that may further contribute to CVD risk (3, 7). In addition, these factors include behaviors, anthropometric conditions, and symptoms and therefore should not be interpreted as a validated behavioral intervention score.

Importantly, previous studies have generally evaluated genetic susceptibility and health-related risk over a single overall follow-up period. It remains unclear whether inherited susceptibility and health-related factors show different risk patterns during earlier and later periods of CVD development among adults with depression. Inherited susceptibility may be more evident in near-term risk, whereas the cumulative influence of behavioral, anthropometric, sleep-related, and cardiometabolic factors may become more apparent over longer follow-up.

We therefore examined the joint associations of CVD PRS and a health-related risk profile with incident CVD among UK Biobank participants with depression. We separately evaluated risk during years 0–5 after the person-specific analytic index and during years 5–10 using a landmark analysis. Our objective was to characterize joint and temporal risk patterns rather than to determine whether health-related factors causally offset inherited susceptibility.

## Methods

### Data source and study design

**Figure 1:**
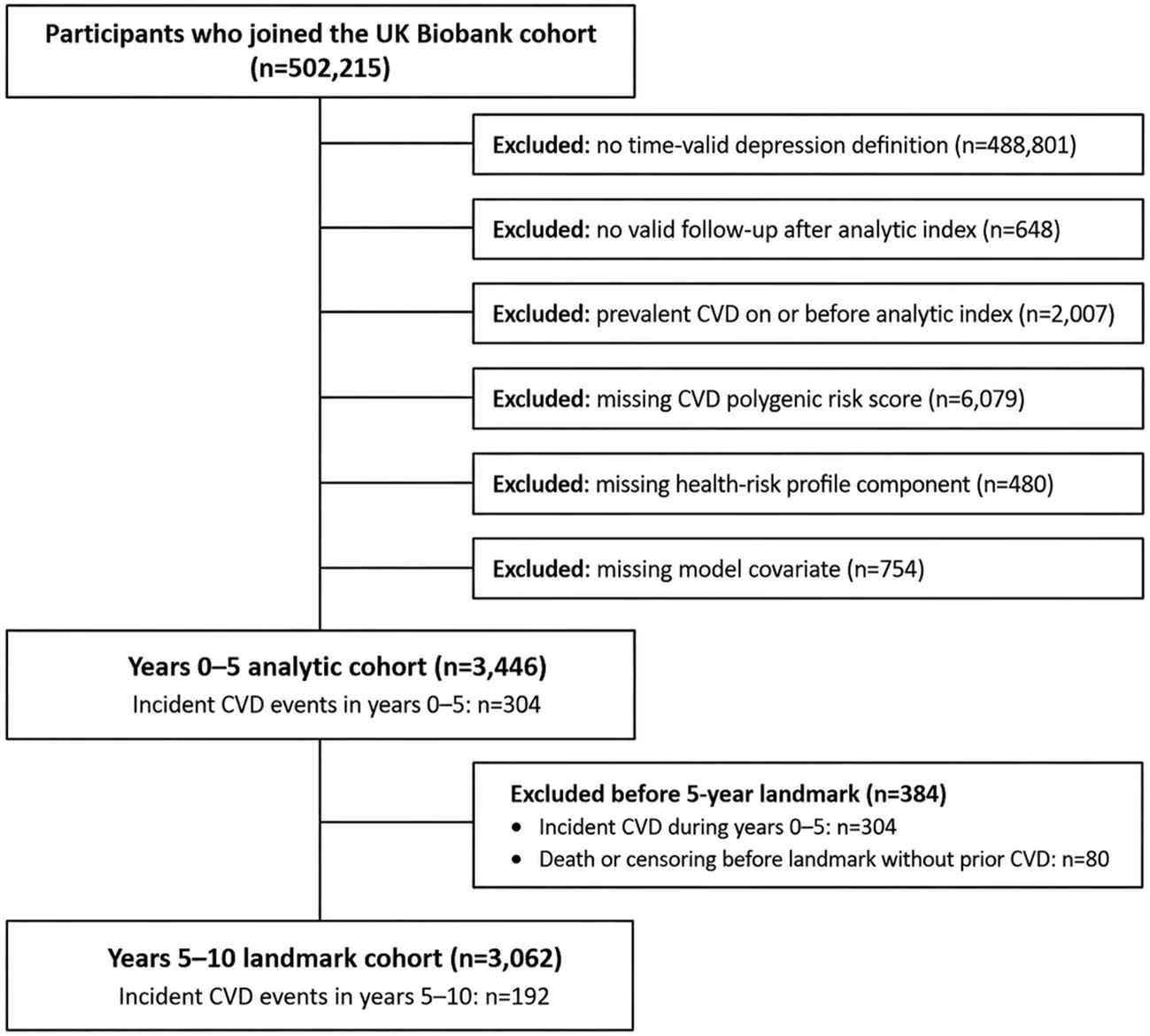
Flow diagram for the selection of UK Biobank participants with depression. Depression was defined using linked ICD-10 diagnosis codes F32–F33 or a Patient Health Questionnaire-9 score of at least 10. Exclusions were applied sequentially and were mutually exclusive.

This study used data from the UK Biobank, a population-based cohort that enrolled approximately 500,000 adults aged 40–69 years across the UK during 2006–2010. It included questionnaire data, physical measurements, biochemical markers, employment information, hospital inpatient records, and mortality data. Follow-up ended at the earliest occurrence of incident CVD, death, the end of available linked follow-up, or the end of the prespecified analysis window. Therefore, with a diverse dataset, many studies have been using the UK Biobank dataset for scientific research (8–11). The UK Biobank received ethics approval from the North West Multi-centre Research Ethics Committee (16/NW/0274), and all participants provided written informed consent (12). Among 502,215 UK Biobank participants, we sequentially excluded 488,801 participants without a time-valid depression definition, 648 without valid follow-up after the person-specific analytic index, 2,007 with prevalent CVD on or before the analytic index, 6,079 without an available CVD PRS, 480 with missing health-related risk-profile components, and 754 with missing model covariates. The resulting complete-case cohort for the years 0–5 analysis comprised 3,446 participants. For the years 5–10 landmark analysis, we additionally excluded 384 participants who experienced incident CVD, died, were censored, or were no longer under observation before the 5-year landmark, leaving 3,062 participants. Participant selection and the analytic timeline are shown in **Figure 1** and **Supplementary Figure 1**, respectively.

### Depression definition and analytic index

Depression was defined using either linked International Classification of Diseases, 10th Revision (ICD-10) diagnosis codes F32–F33 or a Patient Health Questionnaire-9 (PHQ-9) score of at least 10. For participants with an ICD-10 depression diagnosis recorded before recruitment, the analytic index was the recruitment assessment date. For participants identified by PHQ-9, the analytic index was the date of the PHQ-9 assessment at which a score of at least 10 was recorded. Participants who met both the ICD-10 and PHQ-9 definitions were included in the primary cohort and categorized separately in baseline descriptive analyses. Mutually exclusive ICD-10- and PHQ-9-only groups were used for subgroup analyses.

CVD occurring on or before the analytic index was considered prevalent and resulted in exclusion. For participants meeting both the pre-recruitment ICD-10 and PHQ-9 definitions, the PHQ-9 assessment date was used as the analytic index. For participants with a PHQ-9-based index, profile components and most covariates measured at recruitment preceded the index and were carried forward. The mean interval between recruitment and the PHQ-9 analytic index was approximately 7.55 years.

### Incident CVD

The composite CVD outcome included transient ischemic attack (G45), ischemic heart disease (I20–I25), cardiac arrest (I46), atrial fibrillation or flutter (I48), heart failure (I50), cerebrovascular disease (I60–I69), and arterial disease (I70–I79). Incident CVD was defined as the first diagnosis after the analytic index.

The composite was selected to capture a broad spectrum of clinically relevant cardiovascular morbidity. As the included conditions are etiologically heterogeneous, we additionally performed a sensitivity analysis restricted to atherosclerotic cardiovascular disease (ASCVD), defined using ischemic heart disease, ischemic stroke, and arterial disease codes.

### CVD PRS

The analysis used UK Biobank Data-Field 26223, the Standard PRS for cardiovascular disease. This score is part of the UK Biobank Standard PRS Release and was developed using external GWAS training data. In the present study, the score was re-standardized to a mean of 0 and a standard deviation of 1 within the complete-case analytic cohort. It was analyzed continuously per 1-SD increase and categorized into low, intermediate, and high groups according to cohort-specific tertiles (13).

### Health-related risk profile

The health-related risk profile comprised five factors: current smoking, frequent alcohol consumption, obesity, low physical-activity frequency, and insomnia. Each factor was assigned one point. Frequent alcohol consumption was defined as alcohol consumption three to four times per week or more frequently. This definition reflects drinking frequency rather than alcohol quantity, and sex-specific quantity thresholds were not used. Obesity was defined as a BMI of at least 30 kg/m^2^ among non-Asian participants and at least 25 kg/m^2^ among Asian participants. Low physical-activity frequency was defined as a combined frequency of moderate- and vigorous-intensity activity of fewer than 3 days/week. Insomnia was defined as insomnia symptoms sometimes or usually.

Participants with 0–1 factors were classified as having a favorable profile, and those with at least 2 factors were classified as having an adverse profile. Six joint exposure groups were constructed by crossing the three PRS categories with the two profile categories.

### Covariates

Covariates included age, sex, ethnicity, hypertension, diabetes, and dyslipidemia. Ethnicity was grouped as White, Asian, or Other. Hypertension was defined as systolic blood pressure ≥ 140 mmHg, diastolic blood pressure ≥ 90 mmHg, or antihypertensive medication use. Diabetes was defined as glucose ≥ 7.0 mmol/L or antidiabetic medication use. Dyslipidemia was defined using triglycerides ≥ 1.7 mmol/L or lipid-lowering medication use. Definitions of covariates were operationalized using approaches used in prior population-based cohort studies (14–17).

### Statistical analysis

Continuous characteristics are presented as means with standard deviations, and categorical characteristics are presented as numbers with percentages. Differences across the six joint groups were described using *p*-values and average standardized mean differences. Person-years were calculated from the start of each analysis window to the earliest occurrence of incident CVD, death, administrative censoring, or the end of the specified window. Incidence rates and 95% confidence intervals (CIs) were calculated per 1,000 person-years. Cause-specific Cox proportional-hazards models analyzed hazard ratios and 95% CIs. Model 1 included age at the analytic index and sex. Model 2 additionally included ethnicity, hypertension, diabetes, and dyslipidemia. Low PRS plus a favorable profile was the reference joint category. For years 0–5, follow-up began at the person-specific analytic index and ended at the earliest of incident CVD, death, censoring, or 5 years after the index. For years 5–10, eligibility was assessed at 5 years after the analytic index. Participants were required to be alive, CVD-free, uncensored, and under observation at the landmark. Follow-up began at the 5-year landmark and ended at the earliest of incident CVD, death, censoring, or 10 years after the original index. The time scale was reset to zero at the landmark. A global Wald test assessed differences across the six joint categories. The six joint PRS–profile category analyses during the two prespecified follow-up windows were designated as the primary analyses. Interaction, depression-definition subgroup, alternative-profile, individual-component, White-only, ASCVD, multiple-imputation, and model-diagnostic analyses were considered secondary or exploratory. Multiplicative interaction was examined using interaction terms between categorical PRS and profile category and between standardized continuous PRS and the continuous risk-factor count. We also estimated the profile association within each PRS stratum, the continuous PRS association within each profile stratum, and the association per additional risk factor within each PRS stratum. Exploratory additive interaction was described using the relative excess risk due to interaction, attributable proportion due to interaction, and synergy index. Crude nonparametric cumulative-incidence functions were estimated with death before CVD treated as a competing event. Curves were compared using Gray’s test. Missingness was summarized for each variable, and characteristics of included and excluded participants were compared. Multiple imputation was performed for nongenetic covariates using 20 imputations and 20 iterations. PRS, event status, and follow-up time were not imputed. The proportional-hazards assumption was assessed using Schoenfeld residuals. Potential nonlinearity of age and continuous PRS was assessed using spline models, and influential observations were examined using difference in beta (DFBETA). No formal adjustment for multiple comparisons was applied. Therefore, secondary and exploratory analyses were interpreted based on effect estimates and 95% confidence intervals rather than isolated *p*-values. Analyses were conducted using SAS version 9.4 (SAS Institute Inc., Cary, NC, USA) and R version 4.5.2 (R Foundation for Statistical Computing, Vienna, Austria) through RStudio version 2025.09.2 + 418 (Posit Software, PBC, Boston, MA, USA). Nonparametric cumulative-incidence functions and Gray’s tests were estimated using SAS version 9.4. Cox proportional-hazards models and model diagnostics were implemented using the survival package version 3.8–3 and the splines package version 4.5.2. Multiple imputation was performed using the mice package version 3.19.0. The dplyr package version 1.1.4 and readr package version 2.1.6 were used for data management, and ggplot2 version 4.0.1 and patchwork version 1.3.2 were used for graphical presentation.

## Results

### Baseline characteristics

The final complete-case cohort included 3,446 participants. Of these, 1,193 had ICD-10-only depression, 2,062 had PHQ-9-only depression, and 191 had both definitions. The six joint groups included 317 participants with low PRS plus a favorable profile, 831 with low PRS plus an adverse profile, 351 with intermediate PRS plus a favorable profile, 798 with intermediate PRS plus an adverse profile, 350 with high PRS plus a favorable profile, and 799 with high PRS plus an adverse profile.

Participants with an adverse profile had higher prevalences of smoking, frequent alcohol consumption, obesity, low physical activity, and insomnia by definition. Hypertension, diabetes, and dyslipidemia were also generally more frequent in adverse-profile groups. Insomnia was common overall and was prevalent in adverse-profile groups. Detailed characteristics and average standardized mean differences are presented in **Table 1**.

**Table 1:** Baseline characteristics of participants according to joint cardiovascular polygenic risk and health-related risk profile.

Characteristic	Low PRS + favorable (*n* = 317)	Low PRS + adverse (*n* = 831)	Intermediate PRS + favorable (*n* = 351)	Intermediate PRS + adverse (*n* = 798)	High PRS + favorable (*n* = 350)	High PRS + adverse (*n* = 799)	*p*-value	Average SMD
Age (years; SD)	57.88 (8.35)	57.95 (8.02)	58.65 (7.98)	57.89 (7.92)	57.56 (7.86)	57.67 (7.96)	0.496	0.052
BMI (kg/m^2^; SD)	25.97 (3.65)	29.83 (6.06)	25.85 (4.15)	29.78 (6.47)	25.81 (3.67)	30.41 (6.22)	< 0.001	0.498
Number of health-related risk factors (IQR)	1.00 [1.00, 1.00]	2.00 [2.00, 3.00]	1.00 [1.00, 1.00]	2.00 [2.00, 3.00]	1.00 [1.00, 1.00]	2.00 [2.00, 3.00]	< 0.001	1.862
Standardized CVD PRS (SD)	− 0.96 (0.21)	− 0.97 (0.21)	− 0.21 (0.25)	− 0.20 (0.26)	1.23 (0.77)	1.14 (0.73)	< 0.001	2.576
Time from recruitment to analytic index (years; SD)	4.77 (3.64)	5.05 (3.67)	5.23 (3.49)	4.89 (3.66)	5.12 (3.65)	4.72 (3.71)	0.187	0.069
Gender (%)								
Women	226 (71.3)	520 (62.6)	247 (70.4)	512 (64.2)	246 (70.3)	531 (66.5)	0.009	0.094
Men	91 (28.7)	311 (37.4)	104 (29.6)	286 (35.8)	104 (29.7)	268 (33.5)		
Ethnicity (%)								
White	311 (98.1)	798 (96.0)	342 (97.4)	769 (96.4)	326 (93.1)	767 (96.0)	0.084	0.127
Asian	0 (0.0)	11 (1.3)	4 (1.1)	13 (1.6)	8 (2.3)	11 (1.4)		
Other	6 (1.9)	22 (2.6)	5 (1.4)	16 (2.0)	16 (4.6)	21 (2.6)		
Depression source at analytic index (%)								
PHQ-9 only	188 (59.3)	506 (60.9)	229 (65.2)	466 (58.4)	222 (63.4)	451 (56.4)	0.176	0.101
ICD-10 only	114 (36.0)	280 (33.7)	105 (29.9)	280 (35.1)	115 (32.9)	299 (37.4)		
Both PHQ-9 and ICD-10	15 (4.7)	45 (5.4)	17 (4.8)	52 (6.5)	13 (3.7)	49 (6.1)		
Current smoking (%)								
No	311 (98.1)	637 (76.7)	344 (98.0)	608 (76.2)	344 (98.3)	640 (80.1)	< 0.001	0.412
Yes	6 (1.9)	194 (23.3)	7 (2.0)	190 (23.8)	6 (1.7)	159 (19.9)		
Frequent alcohol use (%)								
No	295 (93.1)	438 (52.7)	326 (92.9)	423 (53.0)	315 (90.0)	467 (58.4)	< 0.001	0.589
Yes	22 (6.9)	393 (47.3)	25 (7.1)	375 (47.0)	35 (10.0)	332 (41.6)		
Obesity (%)								
No	297 (93.7)	440 (52.9)	326 (92.9)	426 (53.4)	327 (93.4)	400 (50.1)	< 0.001	0.64
Yes	20 (6.3)	391 (47.1)	25 (7.1)	372 (46.6)	23 (6.6)	399 (49.9)		
Low physical-activity frequency (%)								
No	303 (95.6)	468 (56.3)	337 (96.0)	443 (55.5)	331 (94.6)	431 (53.9)	< 0.001	0.647
Yes	14 (4.4)	363 (43.7)	14 (4.0)	355 (44.5)	19 (5.4)	368 (46.1)		
Insomnia (%)								
No	95 (30.0)	49 (5.9)	111 (31.6)	45 (5.6)	126 (36.0)	37 (4.6)	< 0.001	0.468
Yes	222 (70.0)	782 (94.1)	240 (68.4)	753 (94.4)	224 (64.0)	762 (95.4)		
Hypertension (%)								
No	179 (56.5)	361 (43.4)	197 (56.1)	343 (43.0)	189 (54.0)	335 (41.9)	< 0.001	0.165
Yes	138 (43.5)	470 (56.6)	154 (43.9)	455 (57.0)	161 (46.0)	464 (58.1)		
Diabetes (%)								
No	302 (95.3)	758 (91.2)	339 (96.6)	733 (91.9)	337 (96.3)	719 (90.0)	< 0.001	0.141
Yes	15 (4.7)	73 (8.8)	12 (3.4)	65 (8.1)	13 (3.7)	80 (10.0)		
Dyslipidemia (%)								
No	194 (61.2)	365 (43.9)	205 (58.4)	354 (44.4)	213 (60.9)	363 (45.4)	< 0.001	0.201
Yes	123 (38.8)	466 (56.1)	146 (41.6)	444 (55.6)	137 (39.1)	436 (54.6)		

Values are mean (SD), median [IQR], or *n* (%), as indicated. The analytic index was the recruitment assessment date for participants with ICD-10-only depression recorded before recruitment and the PHQ-9 assessment date for participants with PHQ-9-defined depression, including those who also had prerecruitment ICD-10 depression. CVD PRS was standardized within the complete-case analytic cohort and categorized into tertiles. A favorable profile was defined as 0–1 of five risk factors, and an adverse profile as ≥ 2: current smoking, frequent alcohol use (at least three to four times/week), obesity, low physical-activity frequency, and insomnia. Obesity was defined using BMI ≥ 30 kg/m^2^ in non-Asian participants and ≥ 25 kg/m^2^ in Asian participants. Low physical-activity frequency was based on moderate plus vigorous activity on fewer than 3 days/week. Insomnia was defined as symptoms sometimes or usually. The SMD column is the average standardized mean difference across the six groups.

BMI, body mass index; CVD, cardiovascular disease; ICD-10, International Classification of Diseases, 10th Revision; IQR, interquartile range; PHQ-9, Patient Health Questionnaire-9; PRS, polygenic risk score; SD, standard deviation; SMD, standardized mean difference.

### Joint PRS and profile associations during years 0–5

During years 0–5, 304 incident CVD events occurred. The incidence rate was lowest in the low PRS plus favorable-profile group, at 12.55 per 1,000 person-years, and ranged from 16.18 to 22.97 per 1,000 person-years in the other groups. Compared with low PRS plus a favorable profile, adjusted hazard ratios (aHRs) were 1.37 (95% CI, 0.83–2.27) for low PRS plus an adverse profile, 1.27 (95% CI, 0.70–2.28) for intermediate PRS plus a favorable profile, 1.50 (95% CI, 0.91–2.48) for intermediate PRS plus an adverse profile, 2.00 (95% CI, 1.15–3.47) for high PRS plus a favorable profile, and 1.34 (95% CI, 0.81–2.24) for high PRS plus an adverse profile. The global Wald *p*-value across the six groups was 0.182, and the associations did not follow a monotonic pattern across the joint categories (**Table 2**).

**Table 2:** Incident cardiovascular disease according to joint cardiovascular polygenic risk and health-related risk profile.

Follow-up	CVD PRS + health-related risk profile^a^	Event/Total	PY	IR per 1,000 PY (95% CI)	Model 1 aHR^b^ (95% CI)	*p*-value	Model 2 aHR^c^ (95% CI)	*p*-value
Years 0–5	Low PRS + favorable profile	19/317	1,513.7	12.55 (7.56–19.60)	1.00 (Ref)		1.00 (Ref)	
Years 0–5	Low PRS + adverse profile	75/831	3,929.4	19.09 (15.01–23.93)	1.45 (0.88–2.40)	0.149	1.37 (0.83–2.27)	0.226
Years 0–5	Intermediate PRS + favorable profile	27/351	1,668.3	16.18 (10.67–23.55)	1.23 (0.69–2.22)	0.485	1.27 (0.70–2.28)	0.431
Years 0–5	Intermediate PRS + adverse profile	77/798	3,755.5	20.50 (16.18–25.63)	1.60 (0.97–2.65)	0.065	1.50 (0.91–2.48)	0.116
Years 0–5	High PRS + favorable profile	38/350	1,654.1	22.97 (16.26–31.53)	1.93 (1.11–3.34)	0.020	2.00 (1.15–3.47)	0.014
Years 0–5	High PRS + adverse profile	68/799	3,748.9	18.14 (14.09–23.00)	1.46 (0.88–2.43)	0.143	1.34 (0.81–2.24)	0.258
Years 5–10	Low PRS + favorable profile	12/292	767.0	15.64 (8.08–27.33)	1.00 (Ref)		1.00 (Ref)	
Years 5–10	Low PRS + adverse profile	38/736	1,836.7	20.69 (14.64–28.40)	1.34 (0.70–2.56)	0.381	1.26 (0.65–2.41)	0.495
Years 5–10	Intermediate PRS + favorable profile	15/314	747.4	20.07 (11.23–33.10)	1.31 (0.61–2.81)	0.482	1.33 (0.62–2.84)	0.464
Years 5–10	Intermediate PRS + adverse profile	58/701	1,738.8	33.36 (25.33–43.12)	2.11 (1.13–3.93)	0.019	2.02 (1.08–3.77)	0.028
Years 5–10	High PRS + favorable profile	14/308	772.6	18.12 (9.91–30.41)	1.23 (0.57–2.65)	0.604	1.24 (0.57–2.70)	0.580
Years 5–10	High PRS + adverse profile	55/711	1,788.6	30.75 (23.17–40.03)	2.05 (1.09–3.82)	0.025	1.89 (1.01–3.54)	0.048

The analytic index date was defined as the recruitment assessment date for participants with ICD-10–defined depression recorded before recruitment and as the PHQ-9 assessment date for participants identified by a PHQ-9 score of ≥ 10. For the years 0–5 analysis, follow-up began at the analytic index and ended at the earliest occurrence of incident CVD, death, censoring, or 5 years after the index. The years 5–10 analysis was restricted to participants who were alive, CVD-free, uncensored, and under observation at 5 years after the analytic index. Follow-up began at the 5-year landmark and ended at the earliest occurrence of incident CVD, death, censoring, or 10 years after the analytic index. The global Wald *p*-values comparing the six joint categories were 0.182 for years 0–5 and 0.055 for years 5–10.

a CVD PRS was categorized as low, intermediate, or high according to tertiles of the standardized score in the final analytic cohort. The health-related risk profile comprised current smoking, frequent alcohol use, obesity, low physical-activity frequency, and insomnia, with each factor assigned one point. Frequent alcohol use was defined as alcohol consumption three to four times per week or more frequently. Obesity was defined as BMI ≥ 30 kg/m^2^ in non-Asian participants and BMI ≥ 25 kg/m^2^ in Asian participants. Low physical-activity frequency was defined as a combined frequency of moderate and vigorous physical activity of fewer than 3 days per week. Insomnia was defined as insomnia symptoms sometimes or usually. Participants with 0–1 risk factors were classified as having a favorable profile, and those with ≥ 2 risk factors were classified as having an adverse profile.

b Model 1 estimated adjusted hazard ratios using Cox proportional hazards regression and included age at the analytic index and sex.

c Model 2 estimated adjusted hazard ratios using Cox proportional hazards regression and included age at the analytic index, sex, ethnicity, hypertension, diabetes, and dyslipidemia.

*aHR*, adjusted hazard ratio; *BMI*, body mass index; *CI*, confidence interval; *CVD*, cardiovascular disease; *IR*, incidence rate; *MVPA*, moderate-to-vigorous physical activity; *PRS*, polygenic risk score; *PY*, person-years.

### Joint PRS and profile associations during years 5–10

Among the 3,062 participants eligible at the 5-year landmark, 192 incident CVD events occurred during years 5–10. Compared with low PRS plus a favorable profile, the fully adjusted aHRs were 1.26 (95% CI, 0.65–2.41) for low PRS plus an adverse profile, 1.33 (95% CI, 0.62–2.84) for intermediate PRS plus a favorable profile, 2.02 (95% CI, 1.08–3.77) for intermediate PRS plus an adverse profile, 1.24 (95% CI, 0.57–2.70) for high PRS plus a favorable profile, and 1.89 (95% CI, 1.01–3.54) for high PRS plus an adverse profile. The global Wald *p*-value across the six groups was 0.055 (**Table 2**).

### Subgroup analyses

Among participants with ICD-10-only depression, none of the joint-category estimates during years 0–5 was statistically significant. During years 5–10, intermediate PRS plus an adverse profile was associated with an aHR of 2.20 (95% CI, 1.02–4.72), and high PRS plus an adverse profile was associated with an aHR of 2.22 (95% CI, 1.04–4.77), compared with low PRS plus a favorable profile (**Supplementary Table 1**).

Among participants with PHQ-9-only depression, high PRS plus a favorable profile was associated with higher CVD risk during years 0–5 (aHR, 2.18; 95% CI, 1.06–4.47). No joint category was statistically significantly associated with CVD during years 5–10, and the confidence intervals were wide (**Supplementary Table 2**).

### Cumulative incidence and absolute risk

**Figure 2:**
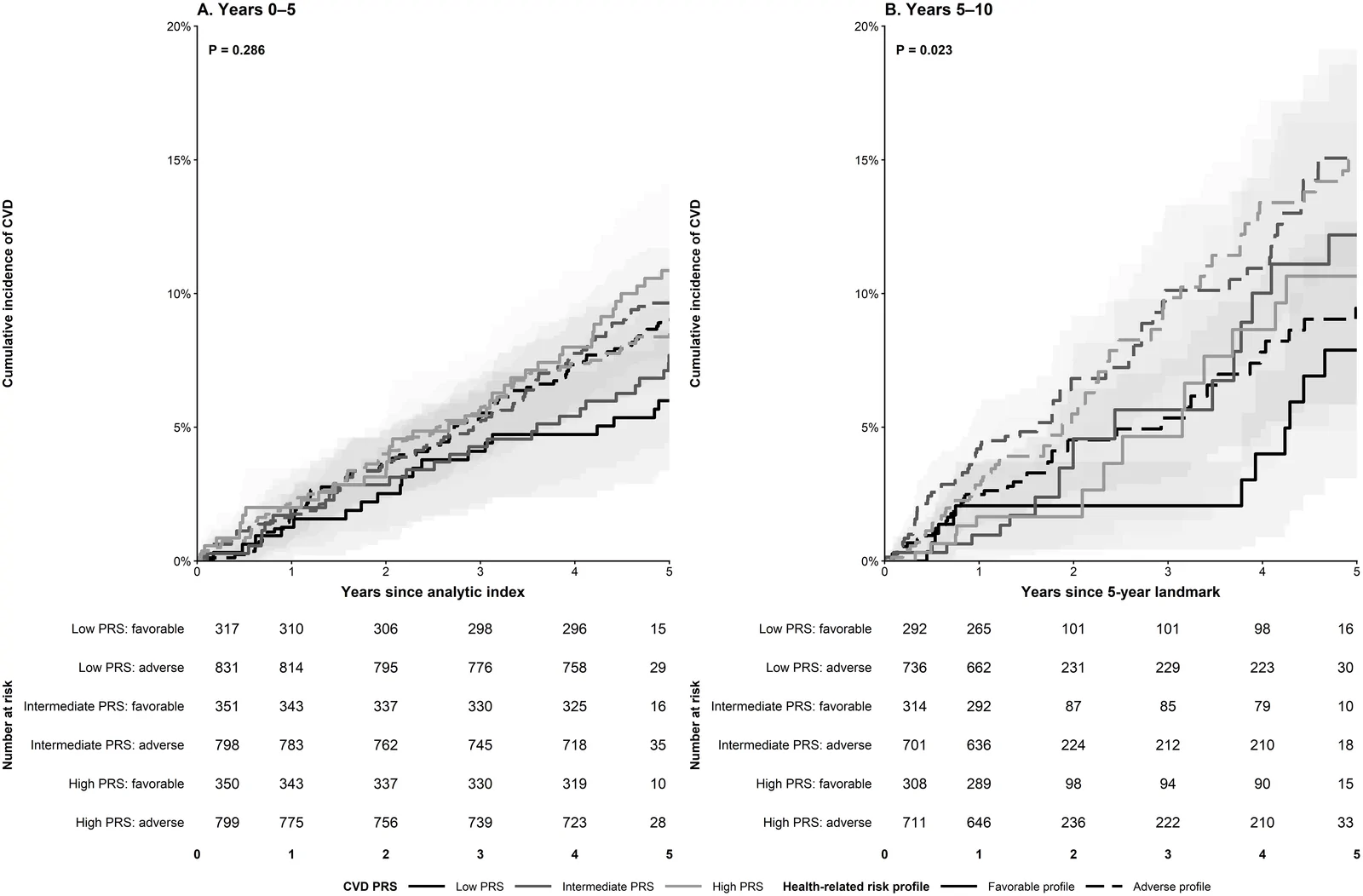
Cumulative incidence of cardiovascular disease according to joint cardiovascular polygenic risk and health-related risk profile among adults with depression. **(A)** The cumulative incidence of cardiovascular disease during years 0–5 after the person-specific analytic index. **(B)** The cumulative incidence during years 5–10 among participants who were alive, cardiovascular disease-free, uncensored, and under observation at the 5-year landmark. The health-related risk profile comprised current smoking, frequent alcohol use, obesity, low physical-activity frequency, and insomnia. A favorable profile was defined as 0–1 of these five factors, whereas an adverse profile was defined as at least two factors. The six groups were formed by cross-classifying low, intermediate, and high cardiovascular polygenic risk score categories with favorable and adverse health-related risk profiles. Cumulative-incidence functions were estimated nonparametrically, treating death before cardiovascular disease as a competing event. Shaded areas indicate pointwise 95% confidence intervals. Gray’s tests compared the cumulative-incidence functions across the six groups. Numbers at risk are presented below each panel at integer follow-up years from year 0 through year 5. CI, confidence interval; CVD, cardiovascular disease; PRS, polygenic risk score.

During years 0–5, crude cumulative CVD risk ranged from 5.99% (95% CI, 3.38%–8.61%) in the low PRS plus favorable-profile group to 10.86% (95% CI, 7.59%–14.12%) in the high PRS plus favorable-profile group. Gray’s test did not demonstrate an overall difference among the six cumulative-incidence functions during this period (*p* = 0.286) (**Figure 2A**; **Supplementary Table 3**).

During years 5–10, conditional cumulative risk among landmark-eligible participants ranged from 7.89% (95% CI, 3.09%–12.68%) in the low PRS plus favorable-profile group to 15.07% (95% CI, 11.00%–19.13%) in the intermediate PRS plus adverse-profile group. Gray’s test indicated differences among the six cumulative-incidence functions (*p* = 0.023). Compared with low PRS plus a favorable profile, the absolute risk differences were + 7.18 percentage points (95% CI, + 0.90 to + 13.46) for intermediate PRS plus an adverse profile and + 7.10 percentage points (95% CI, + 0.81 to + 13.39) for high PRS plus an adverse profile (**Figure 2B**; **Supplementary Table 3**).

### Interaction and within-stratum analyses

The global categorical PRS-by-profile interaction *p*-values were 0.055 during years 0–5 and 0.888 during years 5–10. The continuous standardized PRS-by-continuous-risk-factor-count interaction *p*-values were 0.991 and 0.878, respectively. The association per additional health-related risk factor was not statistically significant in any PRS stratum during either follow-up window (**Table 3**). The adverse-versus-favorable profile associations within individual PRS strata are presented in **Table 3** and **Supplementary Figure 4**. Exploratory additive interaction analyses did not show consistent evidence of positive interaction between high PRS and an adverse profile. During years 0–5, the RERI was − 1.04 (95% CI, − 2.65 to − 0.09), the attributable proportion was − 0.76 (95% CI, − 1.63 to − 0.08), and the synergy index was 0.26 (95% CI, −0.45 to 0.75). During years 5–10, the estimates were imprecise and included their respective null values (**Supplementary Table 4**).

**Table 3:** Multiplicative interaction tests and within-stratum associations of CVD PRS and health-related risk profile with incident CVD.

Measure	Years 0–5	Years 5–10
Categorical PRS tertile × profile, global interaction *p*-value	0.055	0.888
Continuous standardized PRS × continuous risk-factor count, interaction *p*-value	0.991	0.878
Adverse versus favorable profile within low PRS, aHR (95% CI)	1.40 (0.84–2.33)	1.19 (0.61–2.32)
Adverse versus favorable profile within intermediate PRS, aHR (95% CI)	1.14 (0.73–1.78)	1.54 (0.86–2.74)
Adverse versus favorable profile within high PRS, aHR (95% CI)	0.71 (0.47–1.06)	1.51 (0.83–2.75)
CVD PRS per 1-SD increase within the favorable profile, aHR (95% CI)	1.30 (1.07–1.57)	1.20 (0.90–1.60)
CVD PRS per 1-SD increase within the adverse profile, aHR (95% CI)	1.02 (0.89–1.16)	1.13 (0.96–1.32)
Number of health-related risk factors per one-factor increase within low PRS, aHR (95% CI)	1.07 (0.86–1.33)	1.21 (0.90–1.62)
Number of health-related risk factors per one-factor increase within intermediate PRS, aHR (95% CI)	1.09 (0.89–1.33)	1.04 (0.81–1.33)
Number of health-related risk factors per one-factor increase within high PRS, aHR (95% CI)	1.09 (0.89–1.33)	1.14 (0.88–1.47)

The categorical interaction model included CVD PRS tertile, favorable versus adverse health-related risk profile, and their product terms. The reported *p*-value is the 2-*df* global Wald test for the categorical interaction terms. The continuous interaction model included standardized CVD PRS, the continuous count of five health-related risk factors, and their product term. Within-PRS estimates compare the adverse profile with the favorable profile within each CVD PRS category. Within-profile estimates represent the adjusted hazard ratio associated with a 1-SD increase in CVD PRS separately within the favorable and adverse profile strata. All models were adjusted for age at the analytic index, sex, ethnicity, hypertension, diabetes, and dyslipidemia. The continuous risk-factor-count estimates represent the adjusted hazard ratio associated with each additional health-related risk factor separately within the low, intermediate, and high CVD PRS strata. All within-stratum and interaction analyses were considered exploratory.

*aHR*, adjusted hazard ratio; *CI*, confidence interval; *CVD*, cardiovascular disease; *PRS*, polygenic risk score; *SD*, standard deviation.

### Sensitivity analyses

Results varied across alternative definitions of the health-related risk profile, including exclusion of insomnia, exclusion of obesity, use of a behavior-only profile, use of updated BMI, application of an alternative physical-activity definition, and use of an adverse-profile threshold of at least 3 factors during years 0–5. The findings therefore depended partly on the selected components and classification threshold (**Supplementary Table 5**).

None of the individual profile components showed a statistically robust interaction with continuous PRS during either follow-up window (**Supplementary Table 6**). Analyses restricted to White participants and analyses using ASCVD as the outcome produced estimates that were generally directionally consistent with the primary results but were less precise (**Supplementary Table 7**).

### Missingness and model diagnostics

Of 10,759 participants who met the depression criteria, 3,446 were included, and 7,313 were excluded because one or more analytical variables were unavailable. Missing CVD PRS was the principal source of exclusion, affecting 6,079 participants. Variable-specific missingness and comparisons between included and excluded participants are presented in **Supplementary Table 8**.

Multiple-imputation analyses for nongenetic covariates produced results broadly consistent with the complete-case analyses. During years 0–5, the aHR for high PRS plus a favorable profile was 2.04 (95% CI, 1.26–3.30). During years 5–10, the aHRs were 2.17 (95% CI, 1.27–3.73) for intermediate PRS plus an adverse profile and 1.93 (95% CI, 1.12–3.33) for high PRS plus an adverse profile (**Supplementary Table 9**). Multiple-imputation convergence diagnostics are shown in **Supplementary Figures 2**, **S3**.

The global Schoenfeld-residual tests did not indicate violations of the proportional-hazards assumption during years 0–5 (*p* = 0.860) or years 5–10 (*p* = 0.886). Continuous PRS spline analyses showed no evidence of nonlinearity. Age showed evidence of nonlinearity during years 0–5, but age-spline sensitivity analyses produced estimates materially similar to the primary results. DFBETA analyses did not identify extreme influence on the principal interaction estimates (**Supplementary Table 10**).

## Discussion

In this cohort with depression, incident CVD risk differed across joint categories of CVD PRS and a health-related risk profile. During years 0–5, high PRS plus a favorable profile had the largest fully adjusted relative-risk estimate compared with low PRS plus a favorable profile. During years 5–10, intermediate and high PRS combined with an adverse profile had the largest relative and absolute risk estimates. However, the six-category pattern was not monotonic, and formal interaction analyses did not provide consistent evidence of effect modification.

Our results can be considered in relation to previous studies examining genetic and lifestyle risk jointly. Khera et al. found that favorable lifestyle characteristics were associated with substantially lower coronary-event risk within genetic-risk categories (4). Ye et al. also evaluated polygenic and lifestyle factors jointly and found that evidence of interaction varied across cardiovascular and metabolic outcomes (5). In the UK Biobank, Livingstone et al. reported that an unfavorable lifestyle was associated with CVD mortality, myocardial infarction, and stroke independently of genetic risk, while the evidence for gene–lifestyle interaction was not uniform across outcomes (6). Hasbani et al. also showed that more favorable Life’s Simple 7 profiles were associated with lower lifetime coronary heart disease risk across categories of polygenic susceptibility (18). These previous studies support consideration of genetic susceptibility and health-related factors together, but they do not demonstrate that a favorable lifestyle makes genetically distinct groups equivalent.

Although few studies have directly examined whether the relative prominence of genetic susceptibility and health-related risk changes across sequential follow-up periods, related evidence supports such a temporal interpretation. Marston et al. found that a coronary artery disease PRS was more strongly associated with myocardial infarction at younger ages, suggesting that inherited susceptibility may be informative for earlier disease expression (19). Conversely, Chen et al., using repeated assessments of cardiovascular health, showed that subsequent cardiometabolic risk differed according to improvement or deterioration in cardiovascular health, including among individuals with high genetic risk (20). Together, these findings are consistent with fixed genetic susceptibility contributing to earlier vulnerability, while evolving and accumulated health-related factors may become increasingly relevant over time. Our study extends this concept by identifying different descriptive risk patterns during sequential years 0–5 and years 5–10 among adults with depression.

Depression has consistently been associated with increased CVD risk (1–3). Potential pathways include autonomic dysfunction, systemic inflammation, and reduced engagement in health-promoting activities (3, 7). Smoking, obesity, physical inactivity, and sleep disturbance may additionally affect blood pressure and vascular function. Metabolic dysfunction associated with nonalcoholic fatty liver disease has also been closely linked to atherosclerosis and CVD, providing another potential pathway connecting adverse health-related factors with cardiovascular outcomes (21–23). The co-occurrence of depression, inherited susceptibility, and health-related factors may therefore represent overlapping biological and behavioral pathways rather than a simple compensatory relationship.

The depression-definition subgroup findings should also be interpreted cautiously. PHQ-9 assesses depressive symptoms at the time of questionnaire completion, and a score of at least 10 is widely used to identify clinically relevant depressive symptoms (16, 24–26). ICD-10 codes reflect clinically recorded depression but may not capture all participants with current or less severe symptoms. Event numbers were limited in both mutually exclusive subgroup analyses. Differences between the ICD-10-only and PHQ-9-only estimates may therefore reflect differences in depression ascertainment, timing of the analytic index, event numbers, or statistical imprecision rather than true etiologic heterogeneity.

The health-related risk profile was a pragmatic epidemiological classification rather than a validated lifestyle instrument. It combined behaviors with obesity and insomnia, and each component received equal weight. Insomnia was especially common and contributed substantially to adverse-profile classification. Results varied when insomnia or obesity was excluded and when alternative profile definitions were applied. Thus, absence of a consistent composite-profile association should not be interpreted as evidence that individual risk factors are clinically unimportant. Conversely, the observed associations should not be interpreted as the expected effects of interventions targeting all five profile components.

This temporal interpretation should nevertheless be considered hypothesis-generating rather than causal. PRS is fixed from conception, whereas the health-related risk profile was assessed using measurements available at or before the analytic index and was not comprehensively updated during follow-up. We therefore did not directly measure the accumulation or deterioration of risk factors. In addition, PRS and profile status were measured on different scales, and neither the categorical nor continuous interaction analyses showed consistent evidence of interaction. Consequently, the findings do not establish that inherited susceptibility is universally more important than the health-related risk profile or that the relative importance of inherited susceptibility and the profile definitively changes over time. Instead, they suggest that inherited susceptibility may be more evident in near-term CVD risk, whereas health-related risk burden may become more apparent during the later follow-up period among individuals who already have intermediate or high genetic susceptibility.

Several limitations should be considered. First, this observational study is susceptible to residual confounding, reverse causation, and possible overadjustment. Depressive symptoms, preclinical cardiovascular illness, or declining health may also have influenced smoking, physical activity, body weight, sleep, and alcohol frequency, creating the possibility of reverse causation. Hypertension, diabetes, and dyslipidemia may also act as confounders but may also lie on pathways linking the profile components to CVD. Second, depression diagnosis and the analytic index differed between participants. PHQ-9 reflects depressive symptom burden at the time of assessment, whereas linked ICD-10 diagnosis data identify clinically recorded depression. ICD-based depression definitions have been widely used in large-scale administrative and epidemiologic research, and validation studies have generally demonstrated high specificity and positive predictive value, supporting their reliability for identifying clinically recorded cases (25, 27–38). However, their sensitivity is more limited, and milder, untreated, or unrecorded depression may therefore be missed. In addition, for the PHQ-9 group, most profile components and covariates were measured approximately 7.5 years earlier at recruitment and carried forward. Changes during this interval could produce exposure misclassification and regression dilution. Third, the analysis did not comprehensively account for depression duration, recurrence, treatment, symptom trajectories, antidepressant use, anxiety, or other psychiatric comorbidities. Fourth, alcohol was measured according to frequency rather than quantity, and sex-specific quantity thresholds were unavailable. Fifth, the composite CVD outcome included etiologically heterogeneous conditions, and a single CVD PRS may not predict each component equally well. Sixth, missing PRS accounted for most complete-case exclusions, creating potential selection or collider bias despite the broadly consistent multiple-imputation results for nongenetic covariates. Seventh, adjustment for self-reported ethnicity and the White-only sensitivity analysis do not fully address population stratification. Although we used the released UK Biobank Standard CVD PRS, the extracted analytic dataset did not include genetic principal components, genotyping array, genetically inferred ancestry, relatedness information, or SNP-level components of the score. We therefore could not independently evaluate population stratification, ancestry-specific calibration, or variant-level PRS construction. Eighth, UK Biobank participants differ from the general UK population and are subject to healthy-volunteer selection, which may limit generalizability (39). Finally, multiple secondary and sensitivity analyses were performed without multiplicity correction and should be considered exploratory.

In conclusion, among adults with depression, the highest adjusted estimate during years 0–5 was observed in the high-PRS plus favorable-profile group, whereas the highest relative and absolute risk estimates during years 5–10 were observed in the intermediate- and high-PRS groups with adverse profiles. This pattern is consistent with early background vulnerability associated with inherited susceptibility and a later-period association of the health-related risk profile with CVD risk. However, because profile components were not comprehensively updated and formal interaction analyses were not consistently significant, this temporal interpretation remains hypothesis-generating. The findings support joint assessment of inherited susceptibility and the health-related risk profile rather than demonstrating that either is uniformly more important.

## Data Availability

The data used in this study are available from the UK Biobank (https://www.ukbiobank.ac.uk/enable-your-research/apply-for-access). As restrictions apply to the availability of these data, which were used under license for the current study, the authors cannot publicly share these data. This research has been conducted using the UK Biobank Resource under Application Number 106954. Requests to access the datasets should be directed to https://www.ukbiobank.ac.uk/enable-your-research/apply-for-access.
