# Supplementary material for: Joint associations of cardiovascular polygenic risk and a health-related risk profile with incident cardiovascular disease among adults with depression: a UK Biobank study
Source: Front Endocrinol (Lausanne). 2026 Aug 20;17:1934597. doi: 10.3389/fendo.2026.1934597 (PMC13538009; doi:10.3389/fendo.2026.1934597)
Supplement: Supplementary file 1 [file Supplementaryfile1.docx]

**Supplementary Material**

**“Joint Associations of Cardiovascular Polygenic Risk and Health Factors With Incident Cardiovascular Disease Among Adults With Depression: A UK Biobank Study”**

**Table S1.** Incident CVD according to joint CVD PRS and health-risk profile among participants with ICD-10-only depression at the analytic index

**Table S2.** Incident CVD according to joint CVD PRS and health-risk profile among participants with PHQ-9-only depression at the analytic index

**Table S3.** Absolute cumulative risk of CVD and absolute risk differences according to joint CVD PRS and health-risk profile

**Table S4.** Additive interaction between high CVD PRS and an adverse health-risk profile

**Table S5.** Alternative health-risk profile definitions

**Table S6.** Individual health-risk component analyses

**Table S7.** Atherosclerotic cardiovascular disease and White-only sensitivity analyses

**Table S8.** Missingness and included-versus-excluded comparisons

**Table S9.** Multiple-imputation sensitivity analysis

**Table S10.** Model diagnostics and age-spline sensitivity analysis

**Figure S1.** Analytic timeline for depression definition, analytic index, and cardiovascular disease follow-up

**Figure S2.** Multiple-imputation convergence for years 0–5

**Figure S3.** Multiple-imputation convergence for years 5-10

**Figure S4.** Associations of an adverse versus favorable health-risk profile with incident CVD within PRS strata.

**Table S1.** Incident CVD according to joint CVD PRS and health-risk profile among participants with ICD-10-only depression at the analytic index

| **Follow-up** | **CVD PRS + health-risk profile^a^** | **Event/Total** | **PY** | **IR per 1000 PY**  **(95% CI)** | **Model 1 aHR^b^**  **(95% CI)** | ***P* value** | **Model 2 aHR^c^**  **(95% CI)** | ***P* value** |
| --- | --- | --- | --- | --- | --- | --- | --- | --- |
| Years 0-5 | Low PRS + favorable profile | 6/114 | 540.3 | 11.11(4.08-24.17) | 1.00 (Ref) |  | 1.00 (Ref) |  |
|  | Low PRS + adverse profile | 24/280 | 1328.3 | 18.07(11.58-26.89) | 1.65(0.67-4.04) | 0.274 | 1.56(0.63-3.84) | 0.332 |
|  | Intermediate PRS + favorable profile | 8/105 | 503.1 | 15.90(6.87-31.33) | 1.46(0.51-4.20) | 0.486 | 1.45(0.50-4.17) | 0.494 |
|  | Intermediate PRS + adverse profile | 23/280 | 1326.9 | 17.33(10.99-26.01) | 1.56(0.63-3.83) | 0.336 | 1.53(0.62-3.76) | 0.359 |
|  | High PRS + favorable profile | 13/115 | 554.9 | 23.43(12.47-40.06) | 2.15(0.82-5.66) | 0.121 | 2.23(0.85-5.89) | 0.104 |
|  | High PRS + adverse profile | 32/299 | 1368.1 | 23.39(16.00-33.02) | 2.15(0.89-5.15) | 0.087 | 1.99(0.83-4.80) | 0.123 |
| Years 5-10 | Low PRS + favorable profile | 8/103 | 499.4 | 16.02(6.92-31.56) | 1.00 (Ref) |  | 1.00 (Ref) |  |
|  | Low PRS + adverse profile | 28/249 | 1155.5 | 24.23(16.10-35.02) | 1.60(0.73-3.51) | 0.241 | 1.51(0.69-3.34) | 0.304 |
|  | Intermediate PRS + favorable profile | 13/94 | 429.4 | 30.28(16.12-51.77) | 1.99(0.82-4.80) | 0.126 | 1.97(0.82-4.76) | 0.131 |
|  | Intermediate PRS + adverse profile | 40/246 | 1104.9 | 36.20(25.86-49.30) | 2.23(1.04-4.77) | 0.039 | 2.20(1.02-4.72) | 0.043 |
|  | High PRS + favorable profile | 12/101 | 473.7 | 25.33(13.09-44.25) | 1.67(0.68-4.08) | 0.263 | 1.72(0.70-4.22) | 0.237 |
|  | High PRS + adverse profile | 41/257 | 1147.0 | 35.75(25.65-48.49) | 2.37(1.11-5.08) | 0.026 | 2.22(1.04-4.77) | 0.040 |

For the ICD-10-only subgroup, the analytic index date was the recruitment assessment date because eligible ICD-10 depression was recorded before recruitment. For the years 0–5 analysis, follow-up began at the analytic index and ended at the earliest occurrence of incident CVD, death, censoring, or 5 years after the index. The years 5–10 analysis was restricted to participants who were alive, CVD-free, uncensored, and under observation at 5 years after the analytic index. Follow-up began at the 5-year landmark and ended at the earliest occurrence of incident CVD, death, censoring, or 10 years after the analytic index. Participants who also met the PHQ-9 depression definition were not included in this ICD-10-only subgroup analysis.

^a^CVD PRS was categorized as low, intermediate, or high according to tertiles of the standardized score in the final analytic cohort. The health-risk profile comprised current smoking, frequent alcohol use, obesity, low physical-activity frequency, and insomnia, with each factor assigned one point. Frequent alcohol use was defined as alcohol consumption three to four times per week or more frequently. Obesity was defined as BMI ≥30 kg/m² in non-Asian participants and BMI ≥25 kg/m² in Asian participants. Low physical-activity frequency was defined as a combined frequency of moderate- and vigorous-intensity physical activity of fewer than 3 days per week. Insomnia was defined as insomnia symptoms sometimes or usually. Participants with 0–1 risk factors were classified as having a favorable profile, and those with ≥2 risk factors were classified as having an adverse profile.

^b^Model 1 estimated adjusted hazard ratios using Cox proportional hazards regression and included age at the analytic index and sex.

^c^Model 2 estimated adjusted hazard ratios using Cox proportional hazards regression and included age at the analytic index, sex, ethnicity, hypertension, diabetes, and dyslipidemia.

Acronyms: aHR, adjusted hazard ratio; BMI, body mass index; CI, confidence interval; CVD, cardiovascular disease; ICD-10, International Classification of Diseases, 10th Revision; IR, incidence rate; PRS, polygenic risk score; PY, person-years.

**Table S2.** Incident CVD according to joint CVD PRS and health-risk profile among participants with PHQ-9-only depression at the analytic index

| **Follow-up** | **CVD PRS + health-risk profile^a^** | **Event/Total** | **PY** | **IR per 1000 PY**  **(95% CI)** | **Model 1 aHR^b^**  **(95% CI)** | ***P* value** | **Model 2 aHR^c^**  **(95% CI)** | ***P* value** |
| --- | --- | --- | --- | --- | --- | --- | --- | --- |
| Years 0-5 | Low PRS + favorable profile | 11/188 | 904.4 | 12.16(6.07-21.76) | 1.00 (Ref) |  | 1.00 (Ref) |  |
|  | Low PRS + adverse profile | 46/506 | 2393.8 | 19.22(14.07-25.63) | 1.46(0.76-2.83) | 0.258 | 1.39(0.72-2.69) | 0.331 |
|  | Intermediate PRS + favorable profile | 17/229 | 1085.0 | 15.67(9.13-25.09) | 1.23(0.57-2.62) | 0.599 | 1.27(0.59-2.71) | 0.540 |
|  | Intermediate PRS + adverse profile | 45/466 | 2205.6 | 20.40(14.88-27.30) | 1.68(0.87-3.25) | 0.123 | 1.57(0.81-3.05) | 0.181 |
|  | High PRS + favorable profile | 24/222 | 1038.8 | 23.10(14.80-34.38) | 2.13(1.04-4.36) | 0.038 | 2.18(1.06-4.47) | 0.033 |
|  | High PRS + adverse profile | 31/451 | 2147.8 | 14.43(9.81-20.49) | 1.21(0.61-2.41) | 0.590 | 1.13(0.56-2.25) | 0.735 |
| Years 5-10 | Low PRS + favorable profile | 4/176 | 247.7 | 16.15(4.40-41.35) | 1.00 (Ref) |  | 1.00 (Ref) |  |
|  | Low PRS + adverse profile | 7/450 | 631.5 | 11.08(4.46-22.84) | 0.66(0.19-2.25) | 0.502 | 0.61(0.18-2.11) | 0.437 |
|  | Intermediate PRS + favorable profile | 2/207 | 298.7 | 6.70(0.81-24.19) | 0.40(0.07-2.21) | 0.295 | 0.41(0.08-2.26) | 0.309 |
|  | Intermediate PRS + adverse profile | 16/415 | 576.6 | 27.75(15.86-45.06) | 1.74(0.58-5.21) | 0.322 | 1.60(0.53-4.82) | 0.405 |
|  | High PRS + favorable profile | 2/195 | 280.5 | 7.13(0.86-25.75) | 0.49(0.09-2.66) | 0.405 | 0.47(0.09-2.60) | 0.390 |
|  | High PRS + adverse profile | 13/410 | 577.5 | 22.51(11.99-38.49) | 1.41(0.46-4.34) | 0.545 | 1.30(0.42-4.03) | 0.646 |

For the PHQ-9-only subgroup, the analytic index date was the date of the PHQ-9 assessment at which a score of ≥10 was identified. Participants with prevalent CVD before this analytic index were excluded. For the years 0–5 analysis, follow-up began at the analytic index and ended at the earliest occurrence of incident CVD, death, censoring, or 5 years after the index. The years 5–10 analysis was restricted to participants who were alive, CVD-free, uncensored, and under observation at 5 years after the analytic index. Follow-up began at the 5-year landmark and ended at the earliest occurrence of incident CVD, death, censoring, or 10 years after the analytic index. Participants who also met the ICD-10 depression definition were not included in this PHQ-9-only subgroup analysis.

^a^CVD PRS was categorized as low, intermediate, or high according to tertiles of the standardized score in the final analytic cohort. The health-risk profile comprised current smoking, frequent alcohol use, obesity, low physical-activity frequency, and insomnia, with each factor assigned one point. Frequent alcohol use was defined as alcohol consumption three to four times per week or more frequently. Obesity was defined as BMI ≥30 kg/m² in non-Asian participants and BMI ≥25 kg/m² in Asian participants. Low physical-activity frequency was defined as a combined frequency of moderate- and vigorous-intensity physical activity of fewer than 3 days per week. Insomnia was defined as insomnia symptoms sometimes or usually. Participants with 0–1 risk factors were classified as having a favorable profile, and those with ≥2 risk factors were classified as having an adverse profile.

^b^Model 1 estimated adjusted hazard ratios using Cox proportional hazards regression and included age at the analytic index and sex.

^c^Model 2 estimated adjusted hazard ratios using Cox proportional hazards regression and included age at the analytic index, sex, ethnicity, hypertension, diabetes, and dyslipidemia.

Acronyms: aHR, adjusted hazard ratio; BMI, body mass index; CI, confidence interval; CVD, cardiovascular disease; IR, incidence rate; PHQ-9, Patient Health Questionnaire-9; PRS, polygenic risk score; PY, person-years.

**Table S3.** Absolute cumulative risk of CVD and absolute risk differences according to joint CVD PRS and health-risk profile

| **Follow-up** | **CVD PRS + health-risk profile^a^** | **Event/Total** | **Absolute cumulative risk, %**  **(95% CI)** | **Risk difference versus reference, percentage points (95% CI)** |
| --- | --- | --- | --- | --- |
| Years 0-5 | Low PRS + favorable profile | 19/317 | 5.99 (3.38–8.61) | 0.00 (Ref) |
|  | Low PRS + adverse profile | 75/831 | 9.03 (7.08–10.97) | +3.03 (-0.23 to +6.30) |
|  | Intermediate PRS + favorable profile | 27/351 | 7.69 (4.90–10.48) | +1.70 (-2.13 to +5.53) |
|  | Intermediate PRS + adverse profile | 77/798 | 9.65 (7.60–11.70) | +3.66 (+0.33 to +6.98) |
|  | High PRS + favorable profile | 38/350 | 10.86 (7.59–14.12) | +4.86 (+0.68 to +9.05) |
|  | High PRS + adverse profile | 68/799 | 8.51 (6.57–10.45) | +2.52 (-0.74 to +5.77) |
| Years 5-10 | Low PRS + favorable profile | 12/292 | 7.89 (3.09–12.68) | 0.00 (Ref) |
|  | Low PRS + adverse profile | 38/736 | 9.45 (6.18–12.72) | +1.57 (-4.23 to +7.37) |
|  | Intermediate PRS + favorable profile | 15/314 | 12.19 (5.83–18.56) | +4.31 (-3.66 to +12.28) |
|  | Intermediate PRS + adverse profile | 58/701 | 15.07 (11.00–19.13) | +7.18 (+0.90 to +13.46) |
|  | High PRS + favorable profile | 14/308 | 10.65 (4.87–16.44) | +2.77 (-4.74 to +10.28) |
|  | High PRS + adverse profile | 55/711 | 14.99 (10.92–19.06) | +7.10 (+0.81 to +13.39) |

Absolute cumulative risks were estimated using crude nonparametric cumulative-incidence functions, treating death before CVD as a competing event. Years 0–5 estimates represent cumulative incidence during the first 5 years after the person-specific analytic index. Years 5–10 estimates represent conditional cumulative incidence during the 5 years following the landmark among participants who were alive, CVD-free, uncensored, and under observation at the 5-year landmark. These estimates should not be interpreted as unconditional 10-year cumulative risks in the original analytic cohort. Risk differences were calculated by subtracting the cumulative risk in the low PRS + favorable profile group from that in each comparison group and are expressed in percentage points. Confidence intervals were calculated using cumulative-incidence variances for the mutually exclusive groups. Gray’s test for equality of cumulative-incidence functions across the six joint groups yielded P=0.286 for years 0–5 and P=0.023 for years 5–10.

Acronyms: CI, confidence interval; CVD, cardiovascular disease; PRS, polygenic risk score.

**Table S4.** Additive interaction between high CVD PRS and an adverse health-risk profile

| **Measure** | **Years 0–5** | **Years 5–10** |
| --- | --- | --- |
| RERI (95% CI) | −1.04 (−2.65 to −0.09) | 0.37 (−1.02 to 1.21) |
| AP (95% CI) | −0.76 (−1.63 to −0.08) | 0.19 (−0.47 to 0.72) |
| Synergy index (95% CI) | 0.26 (−0.45 to 0.75) | 1.70 (−10.8 to 12.1) |

Additive interaction measures compared high versus low CVD PRS and adverse versus favorable health-risk profiles, using low PRS + favorable profile as the joint reference category. Participants with intermediate PRS were not included in these contrasts. The measures were derived from adjusted Cox-model hazard ratios and should be interpreted as exploratory approximations of additive interaction. Models were adjusted for age at the analytic index, sex, ethnicity, hypertension, diabetes, and dyslipidemia. The null values are 0 for the relative excess risk due to interaction and attributable proportion and 1 for the synergy index.

Acronyms: AP, attributable proportion due to interaction; CI, confidence interval; CVD, cardiovascular disease; PRS, polygenic risk score; RERI, relative excess risk due to interaction.

**Table S5.** Alternative health-risk profile definitions

| **Follow-up** | **Alternative definition** | **Low PRS + adverse** | **Intermediate PRS + favorable** | **Intermediate PRS + adverse** | **High PRS + favorable** | **High PRS + adverse** | **Global P value** |
| --- | --- | --- | --- | --- | --- | --- | --- |
| Years 0–5 | Updated BMI before analytic index | 1.37 (0.83–2.28) | 1.28 (0.71–2.30) | 1.50 (0.91–2.48) | 2.00 (1.15–3.48) | 1.35 (0.81–2.24) | 0.183 |
|  | Excluding insomnia | 0.78 (0.50–1.22) | 0.97 (0.68–1.37) | 1.16 (0.78–1.72) | 1.08 (0.77–1.52) | 1.14 (0.76–1.70) | 0.613 |
|  | Excluding obesity | 1.06 (0.70–1.62) | 1.09 (0.70–1.70) | 1.23 (0.81–1.86) | 1.27 (0.82–1.97) | 1.22 (0.80–1.85) | 0.835 |
|  | Behavior-only profile | 0.77 (0.44–1.33) | 1.02 (0.75–1.39) | 1.32 (0.83–2.10) | 1.11 (0.82–1.51) | 1.27 (0.79–2.06) | 0.555 |
|  | Alternative physical-activity definition | 1.45 (0.81–2.62) | 1.31 (0.65–2.63) | 1.60 (0.89–2.88) | 2.27 (1.19–4.35) | 1.46 (0.81–2.64) | 0.147 |
|  | Adverse profile defined as ≥3 factors | 0.80 (0.51–1.25) | 0.98 (0.70–1.38) | 1.19 (0.80–1.77) | 1.07 (0.77–1.50) | 1.21 (0.81–1.81) | 0.585 |
| Years 5–10 | Updated BMI before analytic index | 1.26 (0.65–2.42) | 1.34 (0.63–2.86) | 2.02 (1.08–3.77) | 1.25 (0.58–2.70) | 1.89 (1.01–3.55) | 0.057 |
|  | Excluding insomnia | 1.67 (0.95–2.94) | 1.92 (1.18–3.12) | 1.96 (1.13–3.39) | 1.74 (1.06–2.86) | 1.93 (1.11–3.37) | 0.126 |
|  | Excluding obesity | 1.14 (0.64–2.03) | 1.50 (0.83–2.68) | 1.81 (1.06–3.10) | 1.29 (0.71–2.34) | 1.78 (1.04–3.06) | 0.119 |
|  | Behavior-only profile | 1.60 (0.86–2.98) | 1.78 (1.18–2.69) | 1.48 (0.76–2.85) | 1.64 (1.08–2.48) | 1.50 (0.74–3.03) | 0.145 |
|  | Alternative physical-activity definition | 1.26 (0.61–2.59) | 1.28 (0.53–3.09) | 2.01 (1.00–4.06) | 1.43 (0.60–3.40) | 1.82 (0.90–3.68) | 0.103 |

Values are adjusted hazard ratios (95% CIs) from Cox proportional hazards models, with low PRS + favorable-profile group as the reference for each alternative definition. Models were adjusted for age at the analytic index, sex, ethnicity, hypertension, diabetes, and dyslipidemia.

Updated BMI used the most recent BMI measurement on or before the analytic index. The behavior-only profile included current smoking, frequent alcohol use, and low physical-activity frequency and excluded obesity and insomnia. The alternative physical-activity definition classified participants as having low physical activity when neither moderate activity ≥5 days/week nor vigorous activity ≥3 days/week was reported. Unless otherwise specified, favorable and adverse profiles were defined as 0–1 and ≥2 risk factors, respectively. The ≥3-factor threshold analysis was conducted for years 0–5 only.

Acronyms: BMI, body mass index; CI, confidence interval; CVD, cardiovascular disease; PRS, polygenic risk score.

**Table S6.** Individual health-risk component analyses

| **health-risk component** | **PRS × component interaction P value, years 0–5** | **PRS × component interaction P value, years 5–10** |
| --- | --- | --- |
| Current smoking | 0.702 | 0.669 |
| Frequent alcohol use | 0.144 | 0.737 |
| Obesity | 0.415 | 0.547 |
| Low physical-activity frequency | 0.066 | 0.944 |
| Insomnia | 0.301 | 0.940 |

Each component was evaluated in a separate Cox model that included continuous standardized CVD PRS, the binary component, and their product term. Models were adjusted for age at the analytic index, sex, ethnicity, hypertension, diabetes, and dyslipidemia. These component-specific analyses were secondary and exploratory.

Acronyms: CVD, cardiovascular disease; PRS, polygenic risk score.

**Table S7.** Atherosclerotic cardiovascular disease and White-only sensitivity analyses

| **Sensitivity analysis** | **Follow-up** | **N** | **Events** | **Low PRS + adverse** | **Intermediate PRS + favorable** | **Intermediate PRS + adverse** | **High PRS + favorable** | **High PRS + adverse** | **Global P value** |
| --- | --- | --- | --- | --- | --- | --- | --- | --- | --- |
| Self-reported White-only | Years 0–5 | 3,313 | 296 | 1.35 (0.81–2.24) | 1.22 (0.68–2.21) | 1.51 (0.91–2.49) | 1.95 (1.12–3.41) | 1.33 (0.80–2.22) | 0.204 |
| Self-reported White-only | Years 5–10 | 2,937 | 186 | 1.29 (0.67–2.48) | 1.34 (0.63–2.86) | 1.96 (1.05–3.68) | 1.31 (0.61–2.85) | 1.83 (0.97–3.45) | 0.126 |
| ASCVD outcome | Years 0–5 | 3,446 | 219 | 1.26 (0.69–2.27) | 1.21 (0.61–2.42) | 1.37 (0.76–2.48) | 2.10 (1.11–3.97) | 1.28 (0.70–2.32) | 0.178 |
| ASCVD outcome | Years 5–10 | 3,062 | 128 | 1.43 (0.62–3.31) | 1.36 (0.51–3.66) | 2.13 (0.95–4.79) | 1.66 (0.64–4.28) | 1.94 (0.86–4.38) | 0.344 |

Values are adjusted hazard ratios (95% CIs) relative to the low PRS + favorable-profile group. Models were adjusted for age at the analytic index, sex, hypertension, diabetes, and dyslipidemia; ethnicity was additionally included where applicable. The White-only analysis was restricted to participants with White ethnicity. The ASCVD sensitivity outcome comprised ischemic heart disease (ICD-10 I20–I25), ischemic stroke (I63), and arterial disease (I70–I79).

Acronyms: ASCVD, atherosclerotic cardiovascular disease; CI, confidence interval; CVD, cardiovascular disease; ICD-10, International Classification of Diseases, 10th Revision; PRS, polygenic risk score.

**Table S8.** Missingness and included-versus-excluded comparisons

| **Characteristic** | **Nonmissing N before complete-case exclusions** | **Missing N (%)** | **Excluded/incomplete (N=7,313)** | **Included complete case**  **(n=3,446)** |
| --- | --- | --- | --- | --- |
| Age at analytic index, years | 10,759 | 0 (0.00) | 58.06 (8.20) | 57.90 (7.99) |
| UK Biobank CVD PRS value before analytic-cohort standardization | 4,680 | 6,079 (56.50) | 0.743 (0.575) | 0.753 (0.565) |
| Men, % | 10,759 | 0 (0.00) | 35.7 | 33.8 |
| White ethnicity, % | 10,699 | 60 (0.56) | 94.7 | 96.1 |
| Current smoking, % | 10,699 | 60 (0.56) | 17.9 | 16.3 |
| Frequent alcohol use, % | 10,700 | 59 (0.55) | 33.8 | 34.3 |
| Obesity, % | 10,680 | 79 (0.73) | 33.9 | 35.7 |
| Low physical-activity frequency, % | 9,843 | 916 (8.51) | 29.0 | 32.9 |
| Insomnia, % | 10,726 | 33 (0.31) | 86.6 | 86.6 |
| Hypertension, % | 10,204 | 555 (5.16) | 45.1 | 53.5 |
| Diabetes, % | 9,178 | 1,581 (14.69) | 5.5 | 7.5 |
| Dyslipidemia, % | 10,097 | 662 (6.15) | 45.1 | 50.8 |

The missingness denominator was 10,759 participants who satisfied the time-origin, follow-up, and prevalent-CVD eligibility criteria before exclusions for missing CVD PRS, health-risk profile components, or model covariates. The complete-case analysis included 3,446 participants; 7,313 eligible participants were excluded because of incomplete genetic, profile, or covariate information. Continuous variables are presented as mean (SD), and categorical variables are presented as percentages among participants with nonmissing data. For CVD PRS, the excluded-group summary was based on the 1,234 excluded participants with an available score.

Acronyms: CVD, cardiovascular disease; PRS, polygenic risk score; SD, standard deviation.

**Table S9.** Multiple-imputation sensitivity analysis

| **Follow-up** | **Joint CVD PRS + health-risk profile group** | **Pooled aHR (95% CI)** | **P value** |
| --- | --- | --- | --- |
| Years 0–5 | Low PRS + favorable profile | 1.00 (Ref) |  |
| Years 0–5 | Low PRS + adverse profile | 1.61 (1.03–2.49) | 0.035 |
| Years 0–5 | Intermediate PRS + favorable profile | 1.28 (0.76–2.16) | 0.361 |
| Years 0–5 | Intermediate PRS + adverse profile | 1.55 (1.00–2.41) | 0.050 |
| Years 0–5 | High PRS + favorable profile | 2.04 (1.26–3.30) | 0.004 |
| Years 0–5 | High PRS + adverse profile | 1.46 (0.93–2.27) | 0.098 |
| Years 5–10 | Low PRS + favorable profile | 1.00 (Ref) |  |
| Years 5–10 | Low PRS + adverse profile | 1.58 (0.91–2.76) | 0.106 |
| Years 5–10 | Intermediate PRS + favorable profile | 1.49 (0.77–2.89) | 0.236 |
| Years 5–10 | Intermediate PRS + adverse profile | 2.17 (1.27–3.73) | 0.005 |
| Years 5–10 | High PRS + favorable profile | 1.28 (0.64–2.56) | 0.477 |
| Years 5–10 | High PRS + adverse profile | 1.93 (1.12–3.33) | 0.019 |

20 imputed datasets were generated using 20 iterations. CVD PRS, outcome indicators, and follow-up times were not imputed.

The five-factor count, favorable/adverse profile, and six joint groups were reconstructed in each completed dataset. Cox models were adjusted for age at the analytic index, sex, ethnicity, hypertension, diabetes, and dyslipidemia, and estimates were pooled using Rubin’s rules. Multiple-imputation convergence plots are presented in Figures S2 and S3.

Acronyms: aHR, adjusted hazard ratio; CI, confidence interval; CVD, cardiovascular disease; PRS, polygenic risk score.

**Table S10.** Model diagnostics and age-spline sensitivity analysis

| **Follow-up** | **Analysis** | **Diagnostic or comparison** | **Estimate or test statistic** | **P value** |
| --- | --- | --- | --- | --- |
| Years 0–5 | Proportional-hazards assessment | Global Schoenfeld-residual test | χ²=5.405; df=10 | 0.860 |
| Years 0–5 | Functional-form assessment | CVD PRS: linear term vs natural spline | LRT χ²=1.493; df=2 | 0.474 |
| Years 0–5 | Functional-form assessment | Age: linear term vs natural spline | LRT χ²=15.70; df=2 | <0.001 |
| Years 0–5 | Influence screening | DFBETA cutoff 0.0341 | Terms exceeding cutoff: CVD PRS and ethnicity levels 2 and 3; PRS × profile max \|DFBETA\|=0.0152 and was not flagged | — |
| Years 0–5 | Age-spline sensitivity | Low PRS + adverse vs low PRS + favorable | aHR 1.38 (0.83–2.29) | 0.209 |
| Years 0–5 | Age-spline sensitivity | Intermediate PRS + favorable vs low PRS + favorable | aHR 1.29 (0.72–2.32) | 0.396 |
| Years 0–5 | Age-spline sensitivity | Intermediate PRS + adverse vs low PRS + favorable | aHR 1.53 (0.92–2.54) | 0.098 |
| Years 0–5 | Age-spline sensitivity | High PRS + favorable vs low PRS + favorable | aHR 2.05 (1.18–3.56) | 0.011 |
| Years 0–5 | Age-spline sensitivity | High PRS + adverse vs low PRS + favorable | aHR 1.37 (0.82–2.28) | 0.229 |
| Years 5–10 | Proportional-hazards assessment | Global Schoenfeld-residual test | χ²=5.079; df=10 | 0.886 |
| Years 5–10 | Functional-form assessment | CVD PRS: linear term vs natural spline | LRT χ²=2.268; df=2 | 0.322 |
| Years 5–10 | Functional-form assessment | Age: linear term vs natural spline | LRT χ²=0.411; df=2 | 0.814 |
| Years 5–10 | Influence screening | DFBETA cutoff 0.0361 | Terms exceeding cutoff: CVD PRS, ethnicity levels 2 and 3, and diabetes; PRS × profile max \|DFBETA\|=0.0191 and was not flagged | — |
| Years 5–10 | Age-spline sensitivity | Low PRS + adverse vs low PRS + favorable | aHR 1.26 (0.65–2.42) | 0.492 |
| Years 5–10 | Age-spline sensitivity | Intermediate PRS + favorable vs low PRS + favorable | aHR 1.32 (0.62–2.83) | 0.468 |
| Years 5–10 | Age-spline sensitivity | Intermediate PRS + adverse vs low PRS + favorable | aHR 2.01 (1.07–3.76) | 0.029 |
| Years 5–10 | Age-spline sensitivity | High PRS + favorable vs low PRS + favorable | aHR 1.24 (0.57–2.68) | 0.588 |
| Years 5–10 | Age-spline sensitivity | High PRS + adverse vs low PRS + favorable | aHR 1.89 (1.00–3.54) | 0.048 |

Proportional hazards were evaluated using Schoenfeld-residual tests. Nonlinearity was assessed by likelihood-ratio comparison of a linear term with a natural spline using 3 degrees of freedom. Age showed evidence of nonlinearity during years 0–5 but not years 5–10; CVD PRS showed no evidence of nonlinearity in either window.

For the age-spline sensitivity analysis, age was modeled using a natural spline with 3 degrees of freedom. The models otherwise included the six-category joint exposure and were adjusted for sex, ethnicity, hypertension, diabetes, and dyslipidemia. The low PRS + favorable-profile group was the reference. Estimates were materially similar to those from the primary linear-age models.

Influence screening used 2/√N as an empirical DFBETA cutoff.

Acronyms: aHR, adjusted hazard ratio; CI, confidence interval; CVD, cardiovascular disease; LRT, likelihood-ratio test; PRS, polygenic risk score.

**Figure S1.** Analytic timeline for depression definition, analytic index, and cardiovascular disease follow-up

For participants meeting both definitions, the PHQ-9 assessment date was used as the analytic index.

**Figure S2.** Multiple-imputation convergence for years 0–5

Trace plots show the chain means of the imputed variables across 20 iterations and 20 imputed datasets. The plots were examined to assess mixing and convergence of the imputation chains. No systematic drift indicating failure of convergence was observed.

**Figure S3.** Multiple-imputation convergence for years 5-10

Trace plots show the chain means of the imputed variables across 20 iterations and 20 imputed datasets. The plots were examined to assess mixing and convergence of the imputation chains. No systematic drift indicating failure of convergence was observed.

**Figure S4.** Associations of an adverse versus favorable health-risk profile with incident CVD within PRS strata.

Adjusted hazard ratios compare participants with an adverse health-risk profile with those with a favorable profile separately within the low, intermediate, and high cardiovascular polygenic risk score categories. Panel A presents associations during years 0–5 after the person-specific analytic index. Panel B presents associations during years 5–10 among participants eligible at the 5-year landmark. Cox proportional hazards models were adjusted for age at the analytic index, sex, ethnicity, hypertension, diabetes, and dyslipidemia. Points indicate adjusted hazard ratios, horizontal lines indicate 95% confidence intervals, and the vertical dashed line indicates a hazard ratio of 1.00.

Abbreviations: CI, confidence interval; CVD, cardiovascular disease; HR, hazard ratio; PRS, polygenic risk score.
